# Editorial: Digital Games and Mental Health

**DOI:** 10.3389/fpsyg.2021.713107

**Published:** 2021-08-18

**Authors:** Rachel Kowert, Christopher J. Ferguson, Paul C. Fletcher, Doris Rusch

**Affiliations:** ^1^Take This, Seattle, WA, United States; ^2^Department of Psychology, Stetson University, DeLand, FL, United States; ^3^Department of Psychiatry, University of Cambridge, Cambridge, United Kingdom; ^4^Department of Game Design, Uppsala University, Uppsala, Sweden

**Keywords:** digital games, mental health, game design, well-being, psychology

Over the last decade, there has been a growing interest in the relationship between mental health and digital games. This discussion has recently returned to the forefront of public discourse with the World Health Organization's (WHO) decision to classify Gaming Disorder (GD) in their new diagnostic manual. While concerns about digital games and mental health often revolve around their potential to exacerbate pre-existing symptomatology (e.g., depression or anxiety), the addition of the GD reflects concerns that games themselves may be detrimental to mental well-being.

The move has also prompted criticisms that the WHO's decision may not be well-grounded in data but rather motivated by politics (the WHO has acknowledged being under pressure from “Asian countries”) or moral panic. Alongside these discussions a new line of research examining how video games can be valuable tools for self-exploration and positively influence mental well-being has also emerged. Through in-game narratives and in-game mechanics, video games are beginning to be recognized as potential tools for fostering psychological growth. These potentials are worthy of examination, in terms of unintentional learning (e.g., fostering psychological growth within games not explicitly designed to do so) as well as intentional game design (e.g., the development of games for mental health).

Interest in the links between mental health and digital games are not limited to player effects but also, game design. Over the last few years, there has been a growing concern about the ways in which mental illness is portrayed in digital game content and how that can impact society's perception of mental health. For example, a 2016 study found 24% of their analyzed games depicted one more mentally ill character, with 69% of them acting violently and in line with a homicidal maniac trope (Shapiro and Rotter, 2016). Notably, the depictions of mental illness in games are not just portrayed through game characters, but settings and game mechanics (e.g., sanity meters) as well (see Dunlap, 2018 for an overview). This has led to growing concerns that stereotyped portrayals of mental illness may contribute to the stigmatization narrative of mental health challenges within society through cultivating new beliefs and/or reinforcing harmful stereotypes. In particular, the stigma that surrounds mental illness has been found to be largely generated and shaped by labeling and stereotyping, which often comes from media messages (Stuart, 2006; Ma, 2017).

These concerns have also, at least partially, contributed to a now a growing community of developers creating games to specifically reflect mental health challenges such as anxiety, depression, and posttraumatic stress disorder (so-called, “deep games”; Rusch, 2017) in more nuanced ways. However, little is known about how the design of these kinds of serious games impacts the developers who create them or the players who engage with them. More research is needed to understand how “deep games,” which are created as spaces of symbolic conflict, liberation, and transformation, impact mental health.

This special issue on Digital Games and Mental Health examined the intersection between mental health and digital games within gaming communities and the gaming industry and aligned across three broad themes: uses and effects research, game design, and game adjacent spaces. The articles in each of these themes are briefly discussed below.

## Uses and Effects Research

There are several articles in this collection that discuss classic uses and effects research for video game use and mental health. This includes an examination of the broader interactions between physical and mental health and their impact on problematic internet use (Chao et al.), what individual and contextual differentiate between beneficial and harmful outcomes within players of the same game (Mandryk et al.) and the impact of game transfer phenomena for dysfunctional playing behaviors (de Gortari and Gackenbach).

Additionally, there are several articles that would fall generally under “uses and effects” that outlined experimental designs where games were used directly as a mental health intervention. This includes work evaluating the impact of exergame based intervention for older adults (Li et al.), the potential for commercial video games as intervention for depression among youths (Poppelaars et al.), games as a direct intervention tool for mental health support (Světlák et al.), how avatar customization in digital games could be used as an intervention tool for anxiety (Pimentel and Kalyanaraman), and the potential for digital games to shift societal stereotypes around mental illness (Ferchaud et al.).

## Game Design

Game design is another area of exploration in this special issue, looking at new frontiers for design as well as discussing challenges and solutions within more traditional design paradigms. This includes examining how existential transformative game design can promote authenticity and tap into games' transformative potential (Rusch and Phelps), a discussion of the challenges of using biofeedback as an intervention in virtual environments (Brammer et al.) and recommendations for implementing gamification for mental health and well-being (Cheng).

## Game Adjacent Spaces

Game adjacent spaces received a significant amount of attention within this special issue. With the rise of online communities and online streaming, these areas of interest continue to grow in importance within the realm of game studies. This includes a discussion of innovative ways to implement crisis intervention and mental health support in game adjacent spaces (Carras et al.), their impact on viewers mental health (de Wit et al.), and how out-of-game factors, such as parental worry, can impact in-game factors (Lieberoth and Fiskaali). Deviant verbal and behavioral actions that take place within games and games adjacent spaces were also evaluated, including the first comprehensive taxonomy of these behaviors (Kowert).

## Concluding Thoughts and Future Directions

We hope that the discussions about digital games and mental health brought to the forefront in this special issue helps to open up broader discussions about digital games as intervention tools, mindful game design, and the uses and effects of games beyond the games themselves. The work within this collection highlights the err in the assumption that games are inherently trivial—-games are an art form that tackle serious subjects and can have significant impact on one's life, including their mental well-being.

Research in this area has historically been mired in presumptions that games are “bad,” particularly the now largely defunct fears about “violence” in such games. The stigmatization of games continue with efforts such as the WHO's “gaming disorder” diagnosis (despite the lack of empirical support). It is important that we set aside these cultural assumptions and better understand the nuances of who may benefit or not benefit from games, under what circumstances, and how some games can be better designed to address the mental health needs of some individuals.

Ultimately, like most media technologies (and art in general), video games are not bad or evil, but how we choose to use them can have a differential impact on differing folks. On balance, we find them to be more likely a source for good than bad and hope that the research included here will help guide those curious about how some games may serve to aid in our understanding of mental health.

## Author Contributions

RK wrote and revised the manuscript. CF, PF, and DR revised the manuscript. All authors contributed to the article and approved the submitted version.

## Conflict of Interest

The authors declare that the research was conducted in the absence of any commercial or financial relationships that could be construed as a potential conflict of interest.

## Publisher's Note

All claims expressed in this article are solely those of the authors and do not necessarily represent those of their affiliated organizations, or those of the publisher, the editors and the reviewers. Any product that may be evaluated in this article, or claim that may be made by its manufacturer, is not guaranteed or endorsed by the publisher.
